# Ambulatory cephalosporin prescribing practices at a freestanding children’s hospital network

**DOI:** 10.1017/ash.2022.300

**Published:** 2022-11-02

**Authors:** Hai Nguyen-Tran, Christine E. MacBrayne, Sarah K. Parker, Nicole M. Poole

**Affiliations:** 1 Division of Infectious Diseases, Department of Pediatrics, University of Colorado School of Medicine, Aurora, Colorado; 2 Department of Pharmacy, Children’s Hospital Colorado, Aurora, Colorado

Antibiotics are prescribed in ∼21% of pediatric ambulatory visits nationally; of those, 16% are broad-spectrum cephalosporins (second–fourth generation).^1^ Broad-spectrum cephalosporins are rarely recommended as first-line therapy for common pediatric infections, except in some instances of penicillin allergy.^2,3^ Cefdinir in particular is frequently prescribed despite its less optimal pharmacokinetics and pharmacodynamics.^4,5^ The overprescription of broad-spectrum cephalosporins leads to the development of antimicrobial resistance, unnecessary cost, and adverse side effects.^6^ Prior studies looking at antibiotic use often solely use electronic medical record (EMR) codes to analyze data; however, limitations exist with this method, and more robust data can be obtained through chart review. In this study, we sought to determine the frequency and indications for broad-spectrum oral cephalosporins prescribed in ambulatory settings at a large, freestanding, children’s hospital network.

## Methods

We conducted a retrospective observational cohort study by EMR chart review of broad-spectrum oral cephalosporins prescribed in ambulatory settings within a freestanding children’s hospital network from January 1 through December 31, 2019, where >750,000 ambulatory encounters occurred. Encounters from the emergency department, urgent care, general, and subspecialty clinics for patients aged ≤26 years with broad-spectrum oral cephalosporin prescriptions (ie, cefdinir, cefixime, cefpodoxime, cefprozil, cefuroxime) were included. Encounters from the hematology–oncology clinic were excluded. Data were stored using REDCap.^7^ Raw data were generated at the Children’s Hospital Colorado. Derived data supporting findings of the study are available from N.M.P. upon request.

Variables collected included age, antibiotic prescribed, antibiotic indication, allergies, recent antibiotic use (within 30 days), and pertinent microbiological culture results. Descriptive statistical analyses were used to evaluate frequency of prescriptions, antibiotic indications, and variables influencing antibiotic choice. This study was exempt from institutional review board review.

## Results

In total, 1,381 encounters were included in the study. The median age was 3.9 years (interquartile range, 1.7–8.25). The most common cephalosporin prescribed was cefdinir (81.3%), followed by cefpodoxime (7.1%), cefprozil (6.6%), cefuroxime (2.8%), and cefixime (2.2%). The most common antibiotic indication was otitis media (67.7%), followed by sinusitis (7.2%), pulmonary infections (6.4%), urinary infections (6.2%), ear, nose, throat infections other than otitis media or pharyngitis (5.1%), multiple diagnoses (3.0%), other diagnoses (eg, skin infections) (2.8%), and pharyngitis (1.5%). Most broad-spectrum cephalosporins were prescribed in the emergency department or urgent-care settings (73.6%). Compared to general care and/or subspeciality clinics, in the emergency department or urgent care, broad-spectrum cephalosporins were prescribed more frequently for acute otitis media (75.7% vs 45.3%) and cefdinir was prescribed less frequently (78.1% vs 90.7% of broad-spectrum cephalosporin prescriptions). Of all encounters, 741 (53.7%) had a penicillin allergy documented; of these, 17.9% were listed ≥5 years preceding the encounter. Penicillin allergy descriptions varied, and most were described as a rash (77.9%). Only 0.5% of allergy descriptions reported anaphylaxis. Recent antibiotic use occurred in 438 (31.7%) encounters. For otitis media (n = 934), penicillin allergy and preceding antibiotic use were common indications for broad-spectrum cephalosporin prescriptions (Table 1). During this period, there were 77 positive cultures, which included urine cultures (83.1%), respiratory cultures (11.7%), and skin and soft tissue cultures (5.2%). Only 15.6% of cultures were resistant to first-generation cephalosporins.

**Table 1.** Broad-Spectrum Cephalosporins Prescribed for Otitis Media and Associated Penicillin Allergy or Recent Antibiotic Use Classified as Acute (New Episode), Recurrent (>1 Episode in the Past 3 Months), and Persistent (Still With Current Episode While on a Course of Antibiotics)

Antibiotic Prescribed	Penicillin Allergy, No. (%)	Preceding Antibiotic in Past 30 Days, No. (%)
**Acute otitis media, new episode (N=520)**		
Cefdinir	365 (70.2)	16 (3.1)
		7 (1.3) Amoxicillin or amoxicillin-clavulanate
		6 (1.2) Cephalosporin^a^
		2 (0.4) Other^b^
		1 (0.2) Unknown
Cefixime	1 (0.2)	0 (0.0)
Cefpodoxime	20 (3.8)	3 (0.6)
		2 (0.4) Other^b^
		1 (0.2) Unknown
Cefprozil	17 (3.3)	1 (0.2) Amoxicillin
Cefuroxime	4 (0.8)	0 (0.0)
**Recurrent otitis media** (**N=291)**		
Cefdinir	74 (25.4)	161 (55.3) 111 (38.1) Amoxicillin or amoxicillin-clavulanate
		20 (6.9) Cephalosporin^a^
		3 (1.0) Other^b^
		19 (6.5) Multiple
		8 (2.7) Unknown
Cefpodoxime	1 (0.3)	2 (0.7)
		1 (0.3) Amoxicillin-clavulanate
		1 (0.3) Cephalosporin^a^
Cefprozil	5 (1.7)	7 (2.4)
		5 (1.7) Amoxicillin or amoxicillin-clavulanate
		2 (0.7) Cephalosporin^a^
Cefuroxime	1 (0.3)	1 (0.3) Multiple
**Persistent otitis media (N=123)**		
Cefdinir	58 (47.2)	111 (90.2)
		103 (83.7) Amoxicillin or amoxicillin-clavulanate
		3 (2.4) Cephalosporin^a^
		3 (2.4) Other^b^
		2 (1.6) Unknown
Cefixime	1 (0.8)	1 (0.8) Amoxicillin
Cefpodoxime	2 (1.6)	3 (2.4)
		2 (1.6) Amoxicillin
		1 (0.8) Cephalosporin^a^
Cefprozil	2 (1.6)	4 (3.3)
		1 (0.8) Amoxicillin-clavulanate
		2 (1.6) Cephalosporin^a^
		1 (0.8) Other^b^
Cefuroxime	1 (0.8)	2 (1.6) Amoxicillin or amoxicillin-clavulanate

a Cephalosporins included cefdinir, cephalexin, or ceftriaxone.

b Other included clindamycin, azithromycin, erythromycin.

## Discussion

Broad-spectrum cephalosporin prescriptions are prevalent in ambulatory settings, and this study highlights different targets for improved prescribing practices. For some encounters, penicillin allergy, recent antibiotic use, or drug-resistant culture results were not listed, yet a broad-spectrum cephalosporin, instead of a narrower and likely more effective choice, was nonetheless prescribed.

Through chart review, we determined the reasons why particular antibiotics were prescribed as opposed to use of EMR codes alone. EMR codes can be misclassified and do not explain the decision-making process of providers. The use of chart review strengthens the results of our study and provides more insight into areas to specifically target for improved prescribing practices.

Cefdinir was the most common cephalosporin prescribed. Compared to amoxicillin or other cephalosporins, cefdinir is poorly absorbed and has a short half-life, which contributes to inadequate treatment of infections and development of drug-resistant bacterial strains.^4,5^ Therefore, if a broad-spectrum cephalosporin is indicated, use of agents with more favorable pharmacokinetics and pharmacodynamics (eg, cefpodoxime, cefuroxime) is recommended.^4^

Perceived penicillin allergy was a predominant reason for broad-spectrum cephalosporin prescriptions, suggesting allergy delabeling as an improvement target. Although ∼10% of US patients report a penicillin allergy, <1% have a true allergic reaction.^8^ Allergy descriptions in this study varied and provide an opportunity to further investigate. Rash without other anaphylaxis criteria may be amenable to rechallenge and prevent unnecessary broad-spectrum cephalosporin prescriptions.^9^

Limitations inherent to retrospective studies include incomplete data that may skew frequencies (eg, missed encounters from verbal orders, allergies removed after encounters or not documented); however, our method of chart review minimizes incomplete information. Other factors difficult to infer from chart review, such as provider preference, pharmacy availability, and insurance coverage, could have contributed to prescription choice. We did not analyze all encounters in which any antibiotic, including broad-spectrum cephalosporins, was prescribed in the study. Finally, antibiotic prescription appropriateness was not assessed (eg, observation instead of antibiotics for otitis media).^10^

Broad-spectrum cephalosporins are frequently prescribed in ambulatory settings but often not indicated. Our findings highlights penicillin allergy labels and cefdinir prescribing as specific targets to improve antibiotic prescribing. Utilization of penicillin delabeling pathways in ambulatory settings, provider education about pharmacokinetics and pharmacodynamics of oral cephalosporins, and point of care electronic prescribing modifications (eg, cefpodoxime suggested instead of cefdinir for penicillin allergy) should be next steps for improved prescribing practices.
